# Persistent eschar-like wound healing after Q-switched 1064 nm hybrid nanosecond-picosecond laser monochromatic tattoo removal: management and evolution

**DOI:** 10.1007/s10103-024-04101-3

**Published:** 2024-06-11

**Authors:** Lasarus Mitrofanoff, Susanna Marini, Leonardo Marini

**Affiliations:** 1 Tohtoripalvelu Itu Oy, Syväraumankatu 2 c 10, Rauma, 26100 Finland; 2 The Skin Doctors’ Center, via dei Bonomo 5/a, Trieste, 34126 Italy

**Keywords:** QS laser, Tattoo removal, Wound healing complications, Post-operative skin care

## Abstract

Q-switched (QS) Nd: YAG lasers are frequently utilised light sources for tattoo removal due to their precise micro-confined photo-acoustic interaction with exogenous and endogenous pigments. In order to achieve optimal results, several treatment sessions are usually required. However, the number of sessions depend on tattoo size, design complexity, pigment quantity within dermal layers, and anatomical location. Higher energy settings have often been used to reduce treatment sessions to a minimum however, this approach may lead to possible post-laser skin complications such as pathological wound healing. This case report highlights the importance of recognizing early stages of pathological wound healing encountered after high fluence 1064 nm QS laser tattoo removal. Early implementation of a proportional wound care strategy with anti-neoangiogenic and anti-inflammatory properties through the unconventional use of potent topical steroids applied in a pulsed fashion resulted in positive control of the tissue repair processes. This approach led to effective wound healing re-modulation achieving near normal skin remodelling and optimal tissue healing which in turn, permitted the completion of necessary QS tattoo removal sessions to accomplish successful and safe tattoo fading whilst maintaining overall patient satisfaction.

## Introduction

Laser tattoo removal with QS nanosecond and picosecond lasers has become increasingly popular in recent years [1, 2]. QS lasers deliver highly concentrated energies that generate extremely precise temperature “mega-loads” within wavelength-specific nanometre-sized micro-targets, effectively minimising temperature dissipation to peripheral tissues. This specific laser-tissue interaction is unique to QS lasers and results in the formation of plasma, responsible for fragmenting micro-targets through a so-called photo-acoustic-lysis phenomenon. In tattoo removal procedures, tattoo pigments, recently found to be stored in macrophages, are physically fragmented and subsequently drained, via the lymphatic system, to regional lymph nodes. Due to this highly specific laser-tissue interaction, photo-thermal complications are minimised.

QS laser tattoo removal was refined over time thanks to constant improvement of laser technology and the introduction of combination treatment protocols. However, literature confirms that not all patients respond equally well to QS laser tattoo removal procedures stressing the importance of a preliminary analysis of several clinical variables [3]. Individual photo-genetic skin types, specific tattoo characteristics such as ink chemical composition, colour intensity and distribution, age and anatomical location deeply influence QS laser parameters and post-treatment aftercare [3, 4]. Khunger et al. (2015) categorised post laser-assisted tattoo removal adverse results into immediate and delayed complications [3]. Delayed complications include pigmentary changes, allergic reactions, paradoxical pigment darkening, persistent residual tattoo, superficial textural changes, and scarring. Nonetheless, most unwanted post-treatment consequences may be prevented if patients follow a rigorous early and late skincare regime aiming to create an ideal wound healing microenvironment thus minimising commonly observed side-effects [5]. However, despite meticulous implementation of specific post-treatment aftercare protocols, abnormal wound healing might occasionally occur. In these rare, yet well recognizable scenarios, personalized, prolonged, specific topical treatments are required to restore a new equilibrium within unbalanced post-treatment microenvironments, regaining control over abnormal wound healing tissue responses leading to optimal outcomes as described in this case report.

## Case presentation

**Fig. 1:**
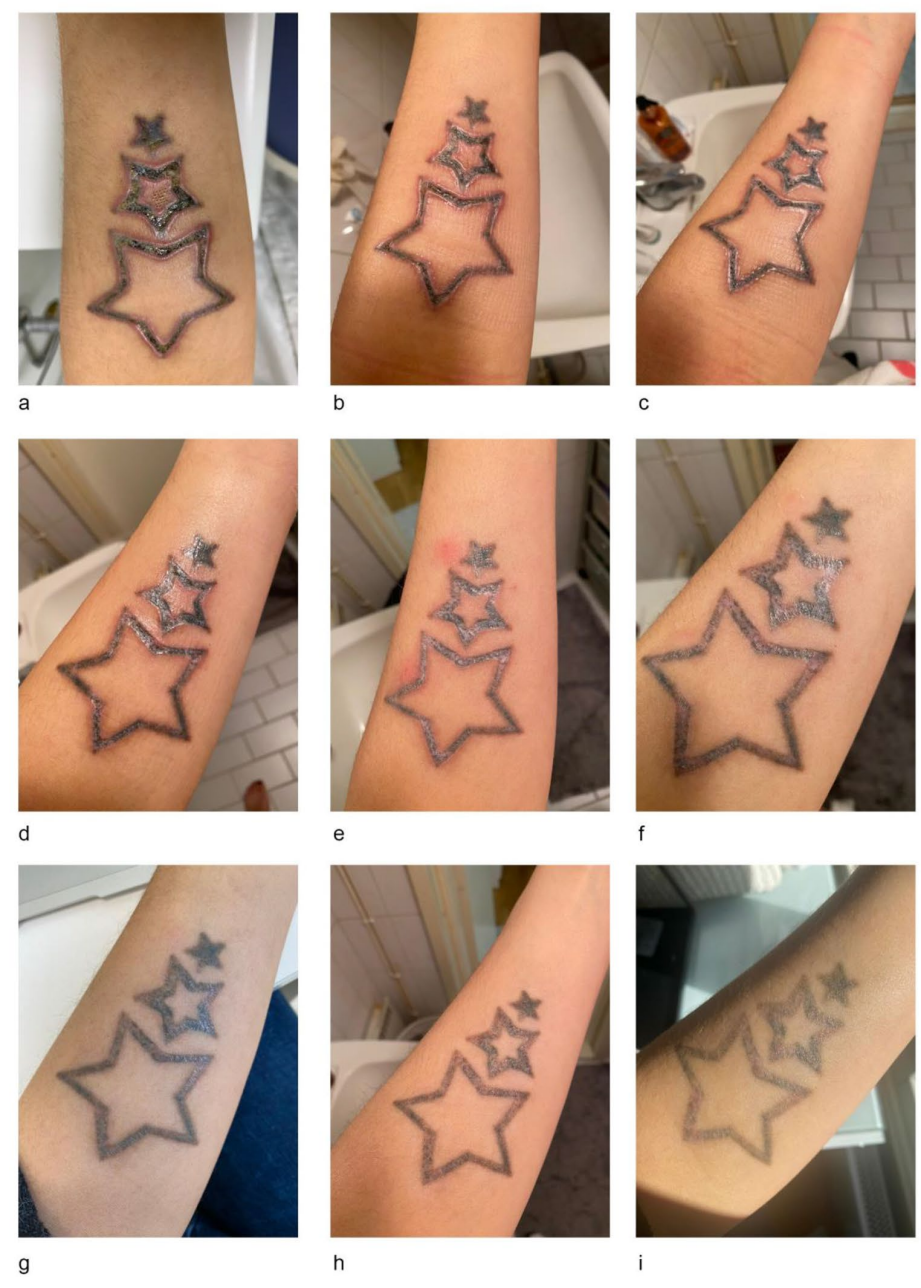
Clinical photographs depicting the wound healing. After: (a) 14 days, (b) 15 days, (c) 16 days, (d) 19 days, (e) 26 days, (f) 33 days, (g) 43 days, (h) 77 days and (i) 106 days, from the first laser tattoo removal session

A 30-year-old lady originally from Karelia with a Fitzpatrick skin phototype III, presented with a black, monochromatic professional tattoo on the medial side of her left forearm. Tattoo was performed 15 years earlier and reported asymptomatic. Patient was otherwise healthy, with a past history of post-inflammatory hyperpigmentation (PIH) on her facial skin after a spot-Jessner peel. A QS 1064-nm, featuring a hybrid nano-picosecond pulse (Starwalker MaQX – Fotona, Slovenia), was proposed to progressively eliminate her tattoo. Our laser-assisted tattoo removal protocol involved a local antisepsis with Neo Amisept (Isopropyl alcohol, Aqua, Didecyl Dimethyl Ammoniumchloride, EDTA) followed by a field block local anaesthesia with 2% lidocaine and 1:200.000 epinephrine. After a 10-min waiting time to allow local vasoconstriction, a first pass with QS 1064 nm Nd: YAG laser in a photo-acoustic fractional mode (9 × 9 mm footprint and 75 mJ/px) was performed. Two full beam laser passes using the same laser system (4 mm round footprint 10% overlap and 4 J/cm^2^) were performed at five-minute intervals. To achieve tissue cooling and gain control over superficial micro-bleeding a sterile gauze soaked in chilled 0.9% sodium chloride was applied over the treated area and covered with ice packs during each 5-minute interval and immediately after the end of the procedure. Post-treatment primary dressing consisted of a topical application of a sterile sheet of Manuka-honey colloidal hydrogel (MediHoney, Integra Life, USA). Precise post-laser treatment instructions were provided recommending a twice-daily removal and application of new Manuka-honey sheet preceded by a 30-min topical application of Aquaphor ointment (Eucerin, Germany) in the morning and 1.5% hydrogen peroxide followed by Cicaplast baume B5 repair crème (La Roche Posay, France) in the evening. Ten days after this skin-care protocol, our patient developed a localized eschar-like tissue alteration (Fig. 1a).

This complication required a re-evaluation of the topical skin care regime introducing the application of anti-neoangiogenic and anti-inflammatory medications. The new regime consisted of three healing phases. Phase I was performed twice a day for 7 days and involved the application of a Class 1 corticosteroid (clobetasol 0.05% ointment) over the whole affected area, followed by the application of a sterile paraffin gauze, a final topical application of dexpanthenol followed by a second sterile paraffin gauze. This topical regime was performed twice a day for 7 days. Clinical appearance improved (Fig. 1e) and topical clobetasol was progressively reduced to three consecutive days per week for 9 weeks and subsequently to two consecutive days per week for 3 more weeks. Aquaphor crème was applied in the morning and Cicaplast Baume B5 crème in the evening when clobetasol was not used. This skin-care regime was rigorously followed for a total of 13 weeks until full re-epithelization, colour normalization, and textural improvement were achieved (Fig. 1i). Full restoration of skin barrier function allowed continuation of QS laser tattoo removal treatments.

**Fig. 2:**
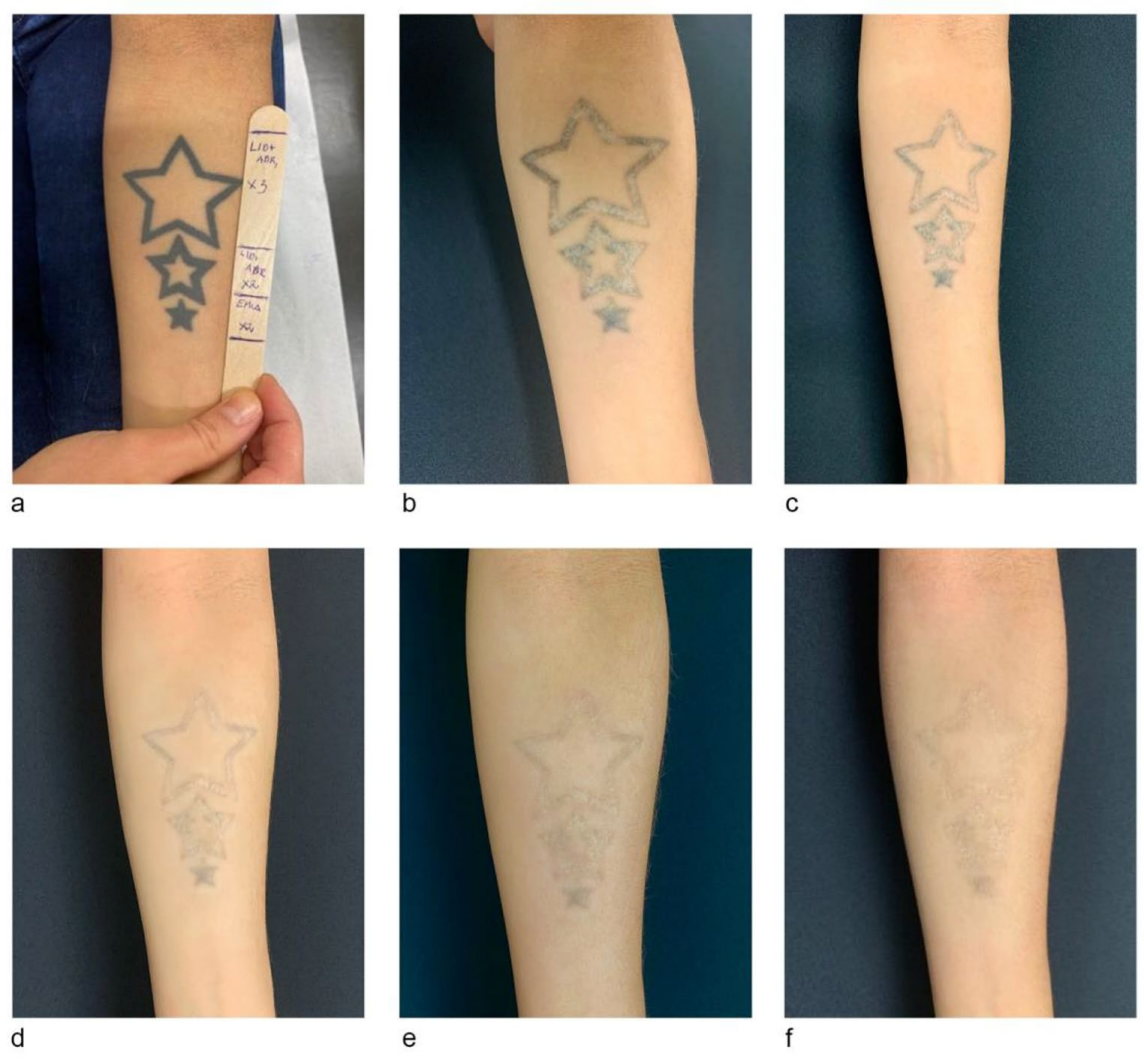
Clinical photographs depicting the healing process after various laser tattoo removal sessions. (a) before treatments, (b) 8 months, (c) 9 months, (d) 13 months, (e) 16 months and (f) 20 months after the first treatment

A total of 6 sessions performed at 2–4-month intervals were necessary to obtain an almost complete tattoo clearing (Fig. 2). The same laser treatment protocol was followed for all subsequent sessions. However, the QS full-beam pass fluence was gradually increased by 1–2 J/cm^2^ between each treatment session tailored according to the progressive tattoo ink fading achieved. Post-treatment healing strategies were maintained constant and consisted of the same topical therapies described after the first laser treatment session. Healing progression and tattoo ink response were documented by standardised digital photographs before each laser session and 4 months after the conclusion of the series of laser treatment (Fig. 2).

## Discussion

Professional tattoo removal with a sequence of three 1064-nm QS nanosecond-picosecond laser passes per session – one fractional mode, followed by two full-beam mode passes at 5-minute intervals, has shown to be an effective and safe treatment protocol to achieve almost full removal of dark and red tattoo inks in fewer sessions than single QS laser pass-per-session treatments [4]. According to the Kirby-Desai tattoo removal scale, which estimates the number of expected laser treatments required to achieve complete tattoo removal according to specific parameters such as patients skin phototype, tattoo anatomical location, colour, amount of ink, presence of scarring and/or tattoo layering, the approximate number of QS laser treatments to obtain complete tattoo fading in this clinical case would have been 11 [6]. However, utilising this innovative combination technique only 6 sessions were required for near complete tattoo fading (Fig. 2, image f).

However, overall outcome is significantly affected by post-treatment skin care regimes. An example of adaptive three stage post-laser skin repair protocol was described by L. Marini et al. in 2022 [4]. One of the most-important aims in effective post-treatment wound healing protocols includes the prevention of pathologic scarring, skin textural chromatic modifications and reduction of post-inflammatory hyperpigmentation.

Normal wound healing consists of four consecutive and overlapping phases [7]. Initially, during the haemostatic phase, clot’s formation alongside local accumulations of cytokines and chemokines attracting neutrophils, monocytes, and T-lymphocytes are the rule. The agglomeration of immune cells within wounded tissues initiates the inflammatory phase. The proliferation phase shortly follows and is characterised by the activation and migration of fibroblasts into wounded areas. Fibroblasts activate to produce neo-collagen, particularly collagen I and III, progressively reconstituting an efficient extracellular matrix. A portion of these activated fibroblasts differentiate into actin-containing myofibroblasts capable of contracting wounds. Simultaneously, epidermal basal cells also migrate to wounded sites and start to proliferate, initiating a re-epithelialisation process to resurface wounded skin. Finally, the remodelling phase induces progressive tissue normalisation and colour uniformity [7, 8].

Our patient developed eschar-like skin alterations one week after her first laser-tattoo removal session despite following precise post-operative skin care instructions. It can be presumed that this exaggerated skin reaction was due to a prolonged inflammatory phase following a set of moderately high laser parameters. Prolonged post-treatment inflammation leads to excessive neo-angiogenesis, fibrosis, and possible abnormal scarring [9]. In order to control this hyperactive inflammatory process, a pulsed regime of potent topical corticosteroids (clobetasol 0.05%) was applied. After approximately 30 days, as seen in the Fig. 1a and i, a complete re-epithelisation was achieved.

The rationale behind the use of pulsed topical application of class 1 corticosteroids is based on their anti-neoangiogenic and anti-inflammatory properties combined with a significant minimization of their tissue atrophying effects. These actions result in long-term beneficial tissue effects such as preventing or reducing PIH during the proliferating and remodelling phases of wound healing. Clinical photographs (Fig. 2b and f) demonstrate an overall successful healing process in the long-run.

Potential risk factors explaining the abnormal modification of post QS laser healing process observed in our case include a darker photogenetic skin type (Fitzpatrick III) and a previous history of PIH reported after a controlled cosmetic chemical peel. Literature confirms darker photogenetic skin type patients are more prone to develop hypo- and hyper-dyspigmentations following accidental or iatrogenic injuries [10]. These individuals are also at higher risk of developing hypertrophic scarring [10]. Additionally, heavily pigmented tattoos, with higher concentrations of localized superficial and deep dermal exogenous pigments, might be at risk of potential complications even if QS lasers are used. This is due to the photo-acoustic laser-tissue interaction in close proximity to the dermal-epidermal junction during the first tattoo removal sessions [11]. In our case, treated tattoo was highly pigmented and a fluence of 4 J/cm^2^, even if considered a relatively safe choice for majority of tattoos, might have been too aggressive, particularly when selected for the first tattoo removal procedure in a darker photo-genetic skin type patient.

Microscopically, the pathologic healing process observed in our patient may have triggered an abnormal, premature activation of myofibroblasts with a consequent, irregular retraction of wounded tissue leading to a less uniform tissue response. Eschar-like wound-healing alterations rapidly responded to continuous and intermittent topical class 1 corticosteroid treatment as demonstrated in Fig. 1c and e. Gradual reduction of topical corticosteroids made treated skin progress towards a smooth and chromatically acceptable skin complexion in 13 weeks.

In conclusion, this case demonstrated the importance of a thorough preliminary clinical evaluation of tattoos before planning a QS laser assisted tattoo removal procedure on which to base an appropriate choice of energy parameters. Dynamic clinical assessment of wound healing processes during early, intermediate, and late post-treatment times are of utmost importance to recognize early signs of abnormal skin repair and initiate properly tailored, personalized skin care regimes. The use of pulsed highly potent topical corticosteroids is an effective strategy to re-route abnormal healing processes towards more controlled skin repair. Personalized, controlled reduction of application frequency schedules has shown to progressively restore a normal healing process resulting in gradual improvement of skin texture and concomitant prevention of PIH.

## References

[1] Cannarozzo G et al (2021) *Q-Switched 1064/532 nm Laser with Nanosecond Pulse in Tattoo Treatment: A Double-Center Retrospective Study* Life (Basel), 11(7) 10.3390/life11070699 PMC8304052 34357071

[2] Gurnani P et al (2022) Comparing the efficacy and safety of laser treatments in tattoo removal: a systematic review. J Am Acad Dermatol 87(1):103–109 32763326 10.1016/j.jaad.2020.07.117

[3] Khunger N, Molpariya A, Khunger A (2015) Complications of tattoos and tattoo removal: stop and think before you ink. J Cutan Aesthet Surg 8(1):30–36 25949020 10.4103/0974-2077.155072 PMC4411590

[4] Marini L et al (2022) Q-S laser micro-drilling and multipass full-beam Q-S laser for tattoo removal - a case series. Lasers Med Sci 37(3):1763–1771 34606037 10.1007/s10103-021-03431-w PMC8971194

[5] Marini L, Odendaal D, Smirnyi S (2017) Importance of Scar Prevention and Treatment-An Approach from Wound Care principles. Dermatol Surg 43(Suppl 1):S85–s90 28009693 10.1097/DSS.0000000000001001

[6] Kirby W et al (2009) The Kirby-Desai Scale: a proposed scale to assess tattoo-removal treatments. J Clin Aesthet Dermatol 2(3):32–37 20729941 PMC2923953

[7] Rodrigues M et al (2019) Wound Healing: a Cellular Perspective. Physiol Rev 99(1):665–706 30475656 10.1152/physrev.00067.2017 PMC6442927

[8] Cañedo-Dorantes L, Cañedo-Ayala M (2019) Skin Acute Wound Healing: a Comprehensive Review. Int J Inflam 2019:p3706315 10.1155/2019/3706315 PMC6582859 31275545

[9] Tripathi S et al (2020) Hypertrophic scars and keloids: a review and current treatment modalities. Biomedical Dermatology, 4

[10] Macedo O, Alster TS (2000) Laser treatment of darker skin tones: a practical approach. Dermatol Ther 13(1):114–126

[11] Naga LI, Alster TS (2017) Laser tattoo removal: an update. Am J Clin Dermatol 18(1):59–65 27722955 10.1007/s40257-016-0227-z

